# A programmable platform for probing cell migration and proliferation

**DOI:** 10.1063/5.0209547

**Published:** 2024-10-29

**Authors:** Jillian Cwycyshyn, Cooper Stansbury, Walter Meixner, James B. Hoying, Lindsey A. Muir, Indika Rajapakse

**Affiliations:** 1 Department of Computational Medicine and Bioinformatics, University of Michigan, Ann Arbor, Michigan 48109, USA; 2 Department of Biomedical Engineering, University of Michigan, Ann Arbor, Michigan 48109, USA; 3 The Michigan Institute for Computational Discovery and Engineering, University of Michigan, Ann Arbor, Michigan 48109, USA; 4 Advanced Solutions Life Sciences, Manchester, New Hampshire 03101, USA; 5 Department of Mathematics, University of Michigan, Ann Arbor, Michigan 48109, USA

## Abstract

The advent of advanced robotic platforms and workflow automation tools has revolutionized the landscape of biological research, offering unprecedented levels of precision, reproducibility, and versatility in experimental design. In this work, we present an automated and modular workflow for exploring cell behavior in two-dimensional culture systems. By integrating the BioAssemblyBot^®^ (BAB) robotic platform and the BioApps™ workflow automater with live-cell fluorescence microscopy, our workflow facilitates execution and analysis of *in vitro* migration and proliferation assays. Robotic assistance and automation allow for the precise and reproducible creation of highly customizable cell-free zones (CFZs), or wounds, in cell monolayers and “hands-free,” schedulable integration with real-time monitoring systems for cellular dynamics. CFZs are designed as computer-aided design models and recreated in confluent cell layers by the BAB 3D-Bioprinting tool. The dynamics of migration and proliferation are evaluated in individual cells using live-cell fluorescence microscopy and an in-house pipeline for image processing and single-cell tracking. Our robotics-assisted approach outperforms manual scratch assays with enhanced reproducibility, adaptability, and precision. The incorporation of automation further facilitates increased flexibility in wound geometry and allows for many experimental conditions to be analyzed in parallel. Unlike traditional cell migration assays, our workflow offers an adjustable platform that can be tailored to a wide range of applications with high-throughput capability. The key features of this system, including its scalability, versatility, and the ability to maintain a high degree of experimental control, position it as a valuable tool for researchers across various disciplines.

## I. INTRODUCTION

Two-dimensional (2D) *in vitro* wound healing assays are frequently used to investigate the dynamics of cellular migration and proliferation.^1,2^ Traditional methods, like the scratch assay or barrier-based assays, are simple and cost-effective but depend on manual handling of tools, equipment, and reagents. This presents challenges such as limited reproducibility, low throughput, and inflexibility in experimental design.^2–4^ Several technologies have been developed to standardize the creation of cell-free zones (CFZs) and address the limitations of manual assays.^4–6^ However, these methods also have their drawbacks. First, uniform CFZs are created in all wells simultaneously or only a few CFZs can be generated at a time,^7,8^ limiting the number of samples and conditions that can be tested in a given experiment. Additionally, versatility and throughput are restricted; different CFZ shapes cannot be produced for multiple samples. To our knowledge, no current technology allows for an adjustable number of conditions and samples without the need for multiple repeated experiments.^5^

We sought to use the BioAssemblyBot (BAB) platform, an intelligent multi-axis robotic system designed for three-dimensional (3D) bio-printing, to explore cell-based assay automation. We present an automated migration and proliferation (MP) assay for cell monolayers by integrating BAB with automated live-cell fluorescence microscopy. Our approach creates precise and reproducible monolayer CFZs, or wounds, and monitors the dynamics of cell behavior in real-time throughout the course of wound closure. Our high-throughput approach enables MP assays to be implemented in any-sized cell culture well plate with the novel ability to generate many differently shaped wounds in a single experiment, allowing for the analysis of multiple conditions in parallel and enhancing versatility in experimental design. We report the development of our automated workflow and demonstrate its advantages in comparison to the standard scratch assay.

## II. RESULTS

### A. Design and automation of migration and proliferation assay

**FIG. 1.**
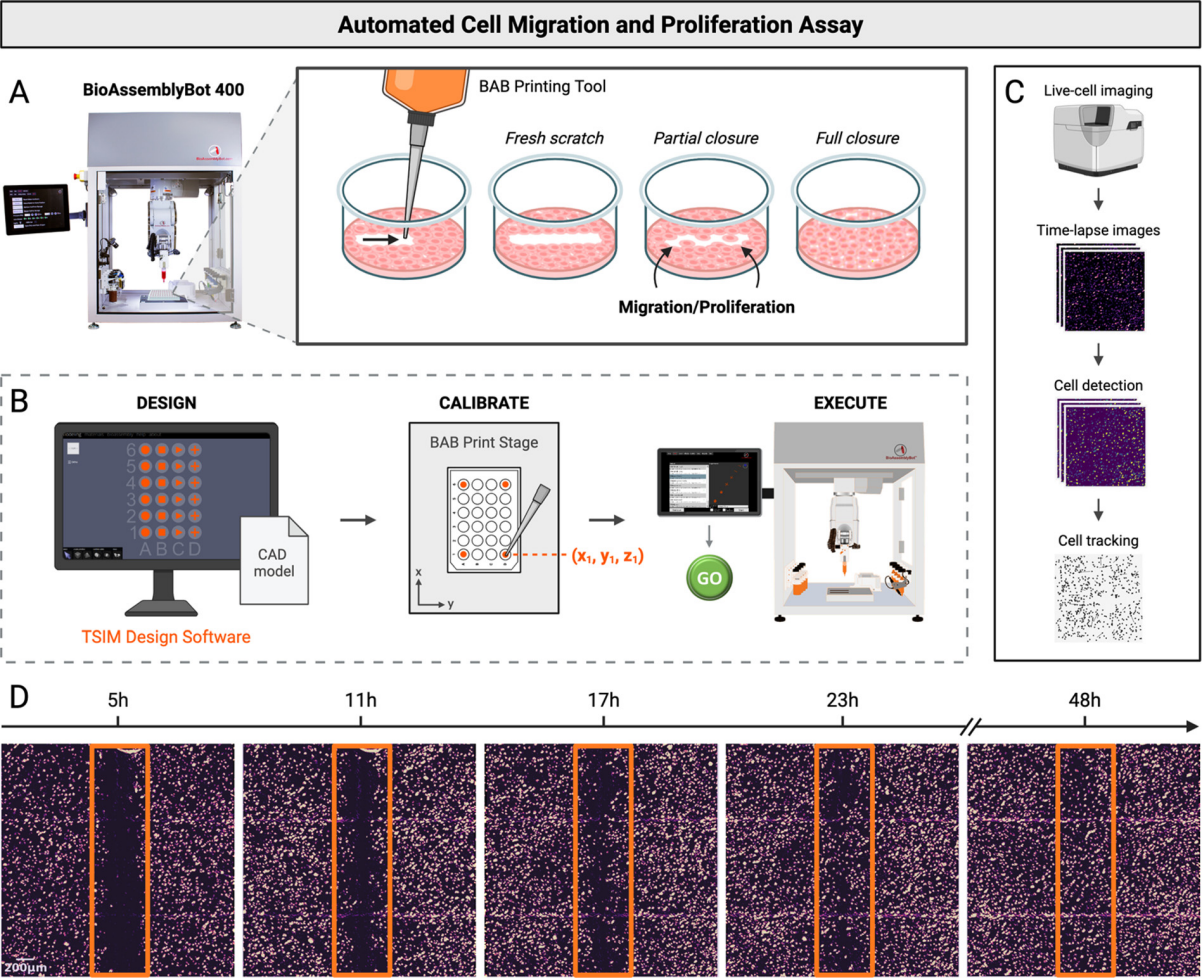
Overview of the automated migration and proliferation assay. The BioAssemblyBot (BAB) platform, including the BAB400 and the Tissue Structure Information Modeling (TSIM) design software, was used for the automation of cell MP assays to increase reproducibility, scalability, and adaptability of experiments. (a) BAB is programmed to mechanically remove user-defined regions from cell monolayers to create a cell-free zone. Processes such as migration and proliferation can be monitored during re-population of the cell-free zone. (b) Workflow to design and create wounds in cell monolayers. First, 3D wound models are designed using TSIM software. Next, the well plate is calibrated with BAB. This involves determining the x, y, and z coordinates of each corner of the plate relative to the print stage with BAB's Printing Tool. Finally, the CAD files are directed to BAB, where the assay is executed in the BAB user interface. (c) Workflow for automated time-lapse image acquisition and analysis. BAB is programmed to transfer the “wounded” well plate to the Zeiss Celldiscoverer7 (CD7) and initiate image acquisition. Images are taken in each well every 20–30 min over the course of wound closure. Individual cells are detected and segmented from each image, and the migration trajectory for each cell is tracked over time. (d) Representative processed images of wound closure over time for a simple scratch wound. Scale bar = 200 *μ*m.

For automated MP assays, we programmed BAB to remove cells with consistent speed and pressure from confluent monolayers using a dispensing tip on its Printing Tool [Fig. 1(a)]. This approach produced highly consistent wound dimensions and minimal damage to the culture surface.

The assay was implemented in three parts: (1) wound design, (2) plate calibration, and (3) assay execution [Fig. 1(b)]. Wounds were designed as computer-aided design (CAD) models using BAB's built-in 3D modeling software. Here, wounds were modeled as 3D shapes flattened onto the culture surface of individual wells in a pre-calibrated well plate, where shape, size, and placement within the wells were customized. Plate calibration was performed on the same type of multi-well plate as the prepared cultures. Coordinates (x–y–z) were programmed for the center of all wells relative to the print stage, and the surface for printing was set to the culture surface of the well plate. The CAD wound models were directed to BAB as a “print” path, and the calibrated plate was selected as the container for printing. The assay was then executed in the BAB user interface, where the wounds were recreated in confluent cell monolayers.

Live-cell imaging enables capture of proliferation and migration dynamics after wounding. As a proof of concept, the automated MP assay was performed on human fibroblasts with simple scratch wounds. We programmed the BAB to transfer the wounded well plate to the Zeiss Celldiscoverer 7 (CD7) live-cell fluorescent microscope with its Pick n' Place tool. Time-lapse images of the cells were captured at 5× magnification every 30 min over 48 h. The acquired images were run through our image processing pipeline, which detects and tracks individual cell nuclei from time-series data [Fig. 1(c)]. Representative images of the simple scratch wound closing over time are depicted in Fig. 1(d).

### B. Automated wounding generates consistent wound dimensions with control over wound shape

**FIG. 2.**
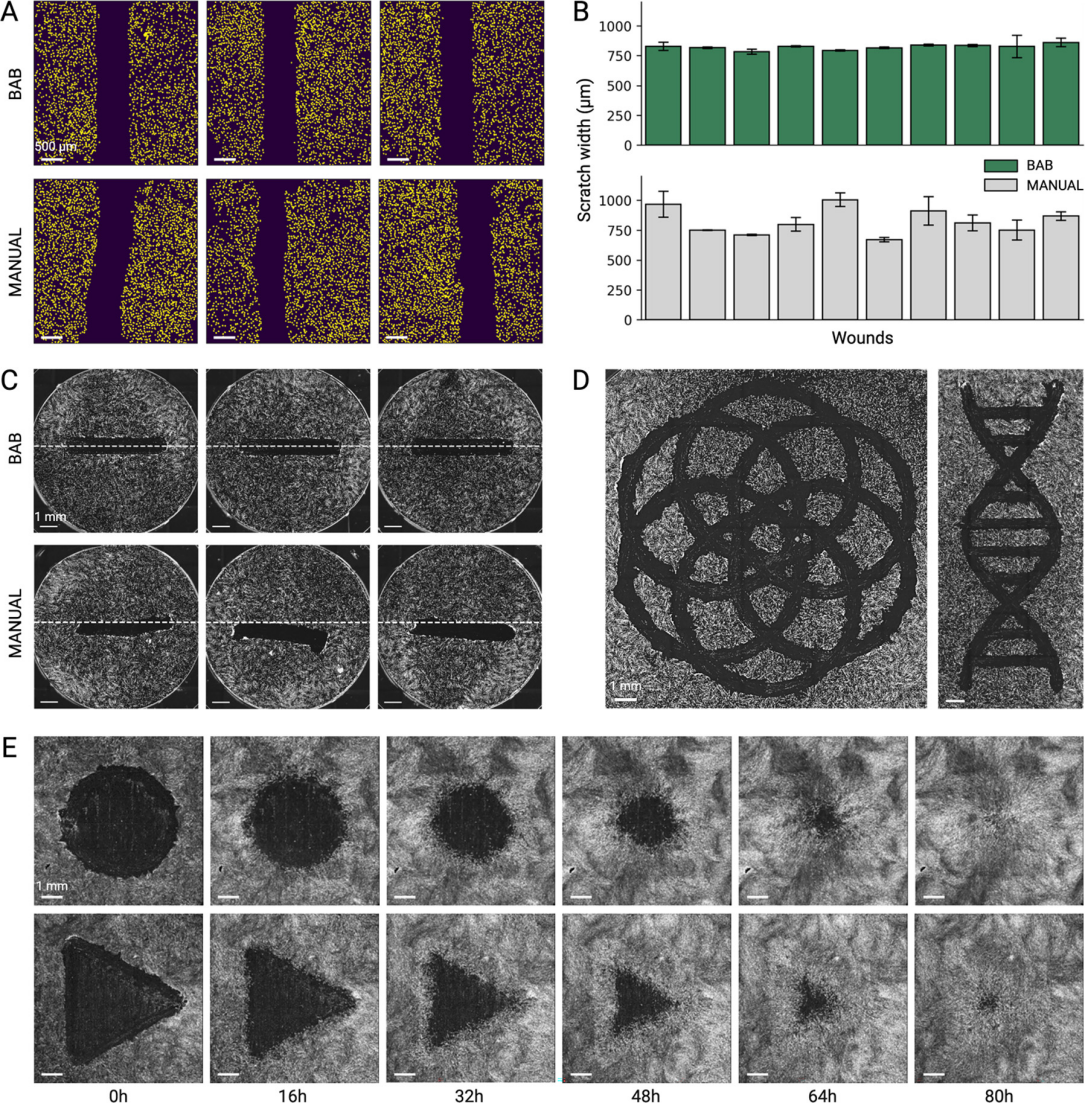
Automated wounding generates more consistent wounds compared to the standard scratch assay. (a) Representative images of simple scratches made by the BAB (top) and by hand (bottom). BAB-generated scratches exhibit more consistency compared to manually generated wounds. Scale bar = 500 *μ*m. (b) Scratch width measurements. Scratch widths and standard deviations were calculated as the distance between each wound edge along the full length of the scratch. Each bar represents one wound. (c) Representative full well images of scratches made by the BAB (top) and by hand (bottom). White dashed lines indicate the center of the well. Scale bar = 1 mm. (d) Representative images of complex wound shapes created by the BAB. Scale bar = 1 mm. (e) Image montage of wound closure over time for circle and triangle wound shapes. Scale bar = 1 mm.

To compare the performance of our automated assay against the standard scratch assay, simple scratches were applied to human fibroblasts by the BAB and manually. Images of each well were taken at 5× magnification, and manual- and BAB-generated scratches were compared for their consistency and positioning. The automated assay produces wounds with increased consistency in wound shape across all wells compared to wounds that were created manually [Fig. 2(a)]. We observed smaller deviations in scratch width for scratches generated by the BAB (*σ* = 44.9 *μ*m) compared to manually generated scratches (*σ* = 103.1 *μ*m) [Fig. 2(b)]. The positioning of scratches across all wells was more variable for manual scratches compared to the automated scratches, which exhibited uniform positioning of each scratch near the center of the well [Fig. 2(c), supplementary material Figs. S1 and S2].

We next tested the impact of tip size, speed, and acceleration on outcomes in the automated assay. Tip sizes with outer diameters (ODs) ranging from 0.3 to 2 mm were used to generate scratches [supplementary material Fig. S3(a)]. Larger tips showed slightly higher variability compared to the smaller tips. The precision metal dispensing tip with an OD of 0.48 mm showed the least variability (*σ* = 33 *μ*m) with an average width of 318 *μ*m per scratch [supplementary material Figs. S3(b) and S3(c)]. In parallel, we evaluated the impact of tip speed and acceleration on scratch consistency. Using a 0.8 mm tip for all scratches, four tip speeds (ranging from 1.5 to 10 mm/s) and three accelerations (ranging from 5 to 20 mm/s^2^) were tested. We observed a minor increase in scratch width with higher speed and acceleration [supplementary material Fig. S3(d)], with the greatest variability occurring at an acceleration of 20 mm/s^2^ [supplementary material Fig. S3(e)].

The BAB's 3D-printing workflow can create many complex wound shapes with high precision [Fig. 2(d)]. This result highlights our ability to consistently generate intricate wounds of various shapes and sizes beyond human capabilities. To evaluate the effect of wound geometry on the rate of closure, we programmed BAB to create four different wound shapes, a triangle, square, circle, and line, and performed live-cell imaging at 20 min intervals over 88 h. Representative images of a circle and triangle wound closing over time are depicted in Fig. 2(e). As expected, the area of the wound had an effect on wound closure time (Table I), but it is undetermined if the effect was due to wound area alone. Each wound shape has a different number of edges, and it has been reported in literature that this can influence wound closure rates.^8^ This emphasizes the importance of reproducibility in cell-based assays for wound healing applications—manually generated wounds with higher error in wound geometry can affect the experimental outcomes. Thus, in a given experiment, wounds need to be carefully designed and created in order to make accurate comparisons within and across different conditions.

**TABLE I.** Average area and wound closure times for different wound shapes (n = 3).

Wound	Area (mm^2^) *±* SD	Closure time	% Closure (*t* = 88 h)
Triangle	21.6 *±* 0.4	*>*88 h	98.8%
Square	17.6 *±* 0.9	*>*88 h	99.9%
Circle	17.2 *±* 1.1	*>*88 h	99.6%
Line	4.4 *±* 0.3	32 h	100%

## III. DISCUSSION

We sought to address the limitations of existing *in vitro* wound healing assays by leveraging the capabilities of the BAB platform. BAB-generated wounds are more consistent in size and positioning compared to manual methods. We demonstrate BAB's ability to create intricate wound shapes at multiple scales, highlighting the precision and flexibility of this approach. Our method offers advantages in terms of reproducibility, scalability, and adaptability. The robotic control of the assay not only minimizes human-induced variability and contamination risks but also ensures consistent wound creation and monitoring. The scalability and adaptability of our platform allows for high-throughput experimentation, making it amenable to multiple experimental conditions and complex wound geometries.

While wound size is a well-established metric in wound healing research, recent literature suggests the importance of wound geometry.^5^ Studies have indicated that the number of wound edges affects cell migration rates both *in vitro* and *in vivo*.^8,9^ Another study showed that cell movement at the wound edge depends on the interplay between local curvature and actin organization, and thus, the geometry of the wound directly influences closure dynamics.^10^ In natural settings, wounds rarely present as simple geometric shapes. Complex wounds, such as those with irregular edges or varying depths, more accurately mimic *in vivo* tissue repair.^11^ While some aspects of these complexities can be explored in 2D models, their relevance becomes more pronounced in 3D culture systems,^1^ where cells interact with their surrounding extracellular matrix and the spatial configuration of wounds can better simulate *in vivo* conditions. Our future work aims to extend our automated workflow to study wound healing dynamics in 3D-bioprinted human skin equivalents. Unlike standard 3D printers, which operate in three planes, BAB utilizes six degrees of freedom to precisely and reproducibly fabricate biological structures. This enhanced mobility allows for complexity beyond the conventional x–y–z planes. In 3D wound healing models, BAB's ability to print at any angle with high resolution allows for the fabrication of intricately patterned tissue constructs and wound shapes that mimic the spatial complexity of *in vivo* wounds. These models would provide critical insights into the influence of wound geometry on healing processes, presenting an opportunity to develop more effective treatment strategies for complex wounds.

**FIG. 3.**
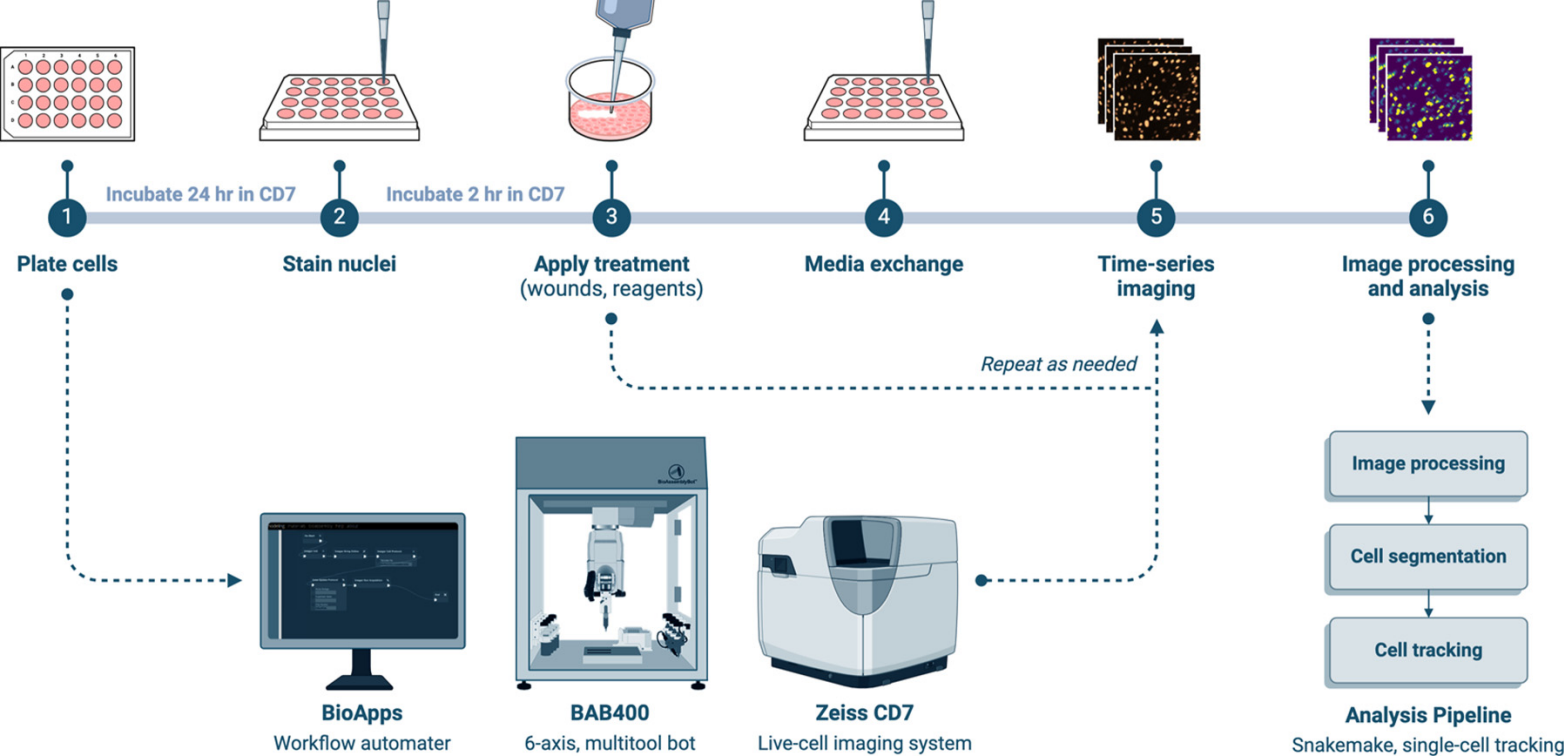
Vision for an end-to-end workflow automated by the BioAssemblyBot platform. All workflow tasks would be assembled into and managed by BioApps, which controls the entire workflow and allows for individual tasks to be tailored as needed.

Beyond 3D-bioprinting, BAB is equipped with interchangeable tools, such as pipetters and grippers, to perform standard laboratory tasks like liquid handling and plate manipulations. This allows for automated media exchanges, reagent delivery, precise cell seeding, and reproducible perturbations, thereby increasing lab efficiency and freeing researchers to focus on other aspects of their work.^12,13^ Through the BioApps workflow automater, BAB can interface with other laboratory equipment to build fully automated workflows. BioApps offers a high degree of programmability, providing control over individual components in a user-friendly interface. This enables customization and flexibility, allowing users to tailor the system to specific experimental needs. We developed a BioApp for automated wounding, plate handling, and time-series image acquisition, available on the BioApps™ Market.^14^ We plan to enhance this BioApp by adding modules for cell plating, fluorescent staining, and media exchanges to provide an end-to-end workflow for automated wound healing assays (Fig. 3). We envision that this workflow can be adapted for any application involving treatments or perturbations within 2D cultures systems.

Our platform's modular design is particularly valuable for instances where experimental design is constrained by human capabilities.^13^ For example, monitoring cell behavior over extended periods involves continuous imaging and media exchanges at regular intervals, which is impractical for manual operation due to the need for consistent timing and minimal disruption to the cells and imaging loci. Likewise, collecting samples at high time resolutions over extended periods would be virtually impossible to perform manually but could be seamlessly managed by the automation capabilities of BAB. Another direction for our future research is automating time-series sample collection for single-cell RNA sequencing during wound healing. We aim to explore gene expression dynamics in single cells, collecting samples every two hours over several days to provide a high-throughput characterization of cellular behavior and molecular changes in response to wounding.

The primary limitation of our approach is the requirement for the BAB and a live-cell imaging system. The initial investment in these technologies could be a barrier for smaller laboratories or those with limited funding. However, the cost of these systems is not entirely out of reach, especially for core services or research facilities prioritizing high-throughput experimentation. In such settings, the benefits of increased efficiency, precision, and reproducibility can justify the investment as they ultimately lead to more reliable and robust scientific outcomes.

We also note that scratch widths were not precisely aligned with the measured tip widths, likely due to differences in tip deformation during scratching that is related to tip material. Material quality could also impact tip performance, where subtle manufacturing inconsistencies might contribute to variation if more than one tip is used. Thus where tip changes are needed, the user should ensure proper calibration for each tip.

In summary, the combined features of BAB and BioApps allow for the automation of end-to-end experimental workflows with increased efficiency, throughput, and standardization. This ensures high precision and accuracy, leading to more reliable and reproducible scientific results. Incorporating live-cell fluorescent imaging in our automated workflow enables the investigation of dynamic biological processes during wound healing, like migration and proliferation, cell cycle phase transitions,^15^ or epithelial-to-mesenchymal transition events,^16^ providing a more comprehensive view of cellular behaviors in response to injury. Our platform is broadly applicable beyond the study of wound healing, including (a) monitoring angiogenesis in bio-fabricated tissue constructs; (b) migration studies with different chemical conditions or coating substrates; (c) *in vitro* models for cancer metastasis and invasion; (d) high-throughput drug screening; and (e) the investigation of cell state trajectories in cellular reprogramming studies.

## IV. CONCLUSION

We have developed an automated workflow for *in vitro* migration and proliferation assays. By addressing the limitations of current methods, we achieved enhanced precision, reproducibility, and flexibility in creating and monitoring cell-free zones. The ability to generate complex wound shapes and scale experiments for high-throughput analysis offers significant advancements for wound healing research. While the initial cost is a barrier, the potential for future enhancements, such as 3D wound models and fine-resolution sample collection, underscores the versatility and transformative impact of our approach. Our modular, automated workflow not only streamlines experimental processes but also paves the way for broader applications in biomedical research.

## V. METHODS

### A. Automated migration and proliferation assay

The steps described below were completed for each run of the automated assay reported in the Results. Multi-well plates from 12–96 wells were tested in our approach. The plate calibration steps were completed once for each plate size used, while the tip to stage offset was determined for each new dispensing tip.

#### 1. Wound design

Tissue Structure and Information Modeling (TSIM) software v1.1.227 (Advanced Solutions Life Sciences, LLC) was used for design of the wounds. The plate type was selected, and settings included “print continuously,” “flatten,” and the printer default for “move between layers.” In a new sketch, the wound shapes were drawn using TSIM's sketching tools. Finalized wound shapes were then copied into other wells as desired for replicates. The z-depth of all objects was set to 0.0001. In the material settings, printing pressure was set to 0 psi, and tip speed and acceleration were set to 3 and 10 mm/s^2^, respectively, unless otherwise noted. The design file was saved and sent as a print job to the BAB human machine interface (HMI).

#### 2. Plate calibration

For plate calibration, multi-well plates were placed on the BAB Print Stage and de-lidded. In the BAB HMI, the BAB Printing Tool was retrieved and a new container for printing was created for each type of plate. The number of rows and columns were input, and the Printing Tool tip was manually positioned in the center of the first well, designated A1, at an arbitrary z coordinate since z coordinates were determined later during tip to stage offset calibration. The x, y, and z coordinates were recorded for A1 and the process was repeated for the remaining three corner wells. The coordinates were then calculated and stored for all wells using the HMI settings Update All Wells (Relative) and Calculate Wells from Extents. Calibration for tip to stage offset ensured contact between the dispensing tip of the BAB Printing Tool and the plate surface. The BAB Printing Tool with a tip was manually positioned to have full contact with the surface of the plate, ensuring no visible deformation of the tip. The measured tip offset was recorded for use during print execution.

#### 3. Execution

The assay was executed using the Print interface of the BAB HMI. The measured tip offset was loaded in the calibration settings alongside the design file received from the TSIM application. The calibrated well plate was selected as the container for printing and the location of the BAB Printing Tool was given.

### B. Cell culture

Human BJ fibroblasts (ATCC CRL-2522) were cultured on standard cultureware in Dulbecco's Modified Eagle's Medium (DMEM, Gibco 11965-092) with 10% Fetal Bovine Serum (FBS, Corning 35-015-CV), 1% MEM Non-Essential Amino Acids (NEAA, Gibco 11140-050), and 1% penicillin-streptomycin (P/S, Gibco 15140122). Cells were incubated at 37 °C in 5% CO_2_, and media were exchanged every 48 h. For the automated MP assay, BJ fibroblasts were seeded at densities ranging from 0.22 to 0.63 × 10^5^ cells/cm^2^ in 12-, 24-, 48-, or 96-well plates. After 24 h, the cells used in time courses were incubated in normal media containing 0.02 *μ*M Hoechst 33342 (Enzo, ENZ-52401) for 2 h, followed by executing the MP assay. Cells used in other assays were stained with 0.5 *μ*M Hoechst for 30 min. After scratching, the wells were washed with PBS. FluoroBrite DMEM (Gibco, A18967-01) with 10% FBS, 1% NEAA, and 1% P/S was added to all wells prior to image acquisition.

### C. Image acquisition

The Zeiss Celldiscoverer 7 (CD7) live-cell imaging system was used to automate capture of time-lapse images during wound closure. Oblique contrast and fluorescence microscopy was performed with a Plan-Apochromat 5×/0.35 objective and 0.5× or 1× tube lens. Images were taken using an Axiocam 506 with 14 bit resolution. Cells were imaged at 37 °C in 5% CO_2_. Images were captured every 20 or 30 min over the duration of wound closure. For each wound, a multi-channel time-series ome.tiff file was prepared in the Zen Blue 3.0 software and exported for downstream analysis.

### D. Image processing

#### 1. Image analysis

Raw images were preprocessed using ImageJ and Python, and analyses were performed with MATLAB and Python. All scripts may be found at the following URL: https://github.com/jrcwycy/wound_healing.

For wound area calculations, raw oblique images were first preprocessed in ImageJ. Wounds were identified using the Image Segmenter application in MATLAB and exported as binary masks. Wound areas were then quantified over time for each shape. For scratch width analysis, individual cell nuclei were segmented from raw H3342 images using StarDist,^17^ a deep-learning based method for object detection and segmentation. Isolated segmentations were then filtered out of the wound bed images using a nearest neighbors approach.^18^ To identify wound edges, contours that separated areas of high cell density from areas of low cell density were detected using scikit-image.^19^ The line of best fit for each contour served as the wound edge for subsequent analyses. Scratch widths were calculated as the distance between the two wound edges along the entire length of the scratch for each image.

#### 2. Automated cell tracking

To automate analysis of wound healing experiments, we constructed an image processing pipeline using the Python framework Snakemake.^20,21^ The pipeline is designed to manage parallel processing of large time-series imaging data in a high-performance computing environment. Briefly, the pipeline produces nuclear segmentations at each time step using StarDist^17^ and predicts cellular movement using a Bayesian single cell tracking approach.^22^ Inputs to the pipeline are multi-channel time-series ome.tiff files and a set of user-defined parameters controlling the behavior of different filtering and analysis operations.^19^ The outputs of the pipeline are properties of cell nuclei at each time step, and nuclear linkages between time steps which we refer to as “tracks.”

We describe the operations of the pipeline in Algorithm 1 on a single input. Note that the pipeline may be run on a set of input images. All software for automated image prepossessing and analysis may be found at the following URL: https://github.com/CooperStansbury/pip-fucci.

**Algorithm 1.** Automated cell tracking.

**Input:** Image **H**_(*c × t×y × x×q*)_ where *c* is the number of color channels, *t* is the number of time steps in the ome.tiff file, *y* is the number of vertical pixels, *x* is the number of horizontal pixels, and *q* is the RGB index.
**Output:** Track table X_(*r × m*)_ where *r* is the number of segmented nuclei times the number of timepoints in their respective tracks and *m* is the number of features for analysis, e.g., estimates of nuclear size, shape, and fluorescent intensity over each color channel.
1: **Flatten:** The RGB channels of image **H** are converted to gray scale resulting in a new image shape **H^¯^**_(*c × t×y × x*)_.
2: **Rescale dimensions:** Image **H^¯^** is rescaled over spatial dimensions *y, x* according to user-defined parameters.
3: **Rescale intensities:** Image **H^¯^** is rescaled over color channels *c* to [0, 255].
4: **Median filtering:** A median filter is applied to **H^¯^** based on user-defined parameters.
5: **Histogram equalization:** Adaptive histogram equalization is applied to **H^¯^** based on user-defined parameters.
6: **Segment:** Image **H^¯^** is segmented using StarDist on the nuclear channel.^17^ Segmentation results are stored as a properties table using skimage.region_props().^19^
7: **Track:** Segmentation results are linked over time using btrack.^22^ The resulting table is merged with the nuclear properties table and stored as *X.*

## SUPPLEMENTARY MATERIAL

See the supplementary material for images and data that support the findings of this study.

## Data Availability

The data that support the findings of this study are available within the article and its supplementary material.
